# P-1039. Cost of CAUTI: A Retrospective Study of 314 catheterized patients

**DOI:** 10.1093/ofid/ofaf695.1234

**Published:** 2026-01-11

**Authors:** Silpita Katragadda, Bismarck Bisono Garcia, Saffa Nadeem, John C O’Horo

**Affiliations:** Mayo Clinic, Rochester, Rochester, Minnesota; Mayo Clinic, Rochester, MN; Mayo Clinic, Rochester, MN; Mayo Clinic, Rochester, MN

## Abstract

**Background:**

Catheter-associated urinary tract infection (CAUTI) is one of the most prevalent nosocomial infection reported and is associated with significant resource expenditure across health care facilities.

**Figure:**
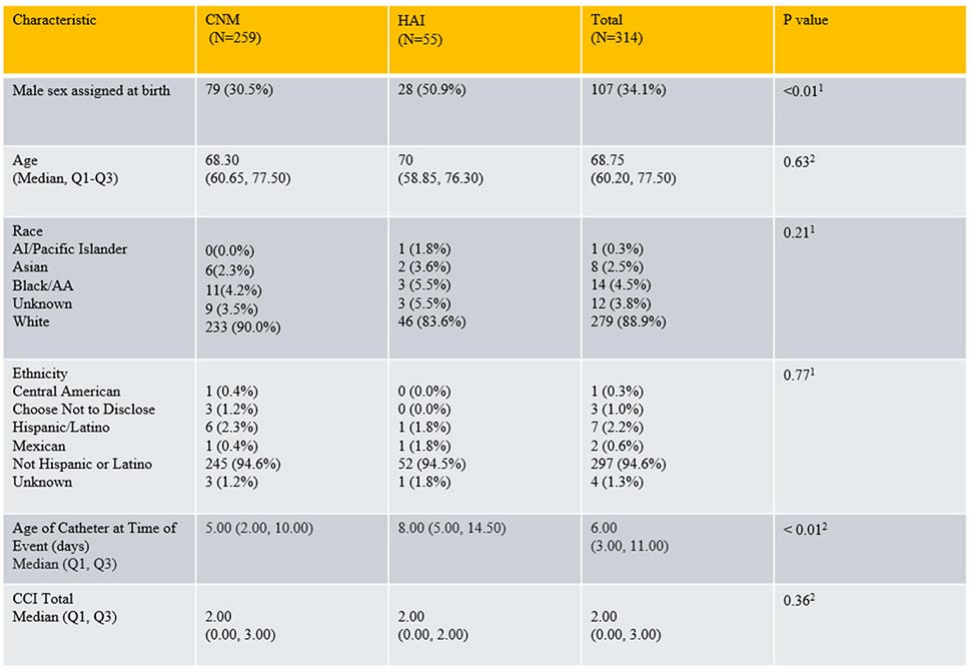

**Figure:**
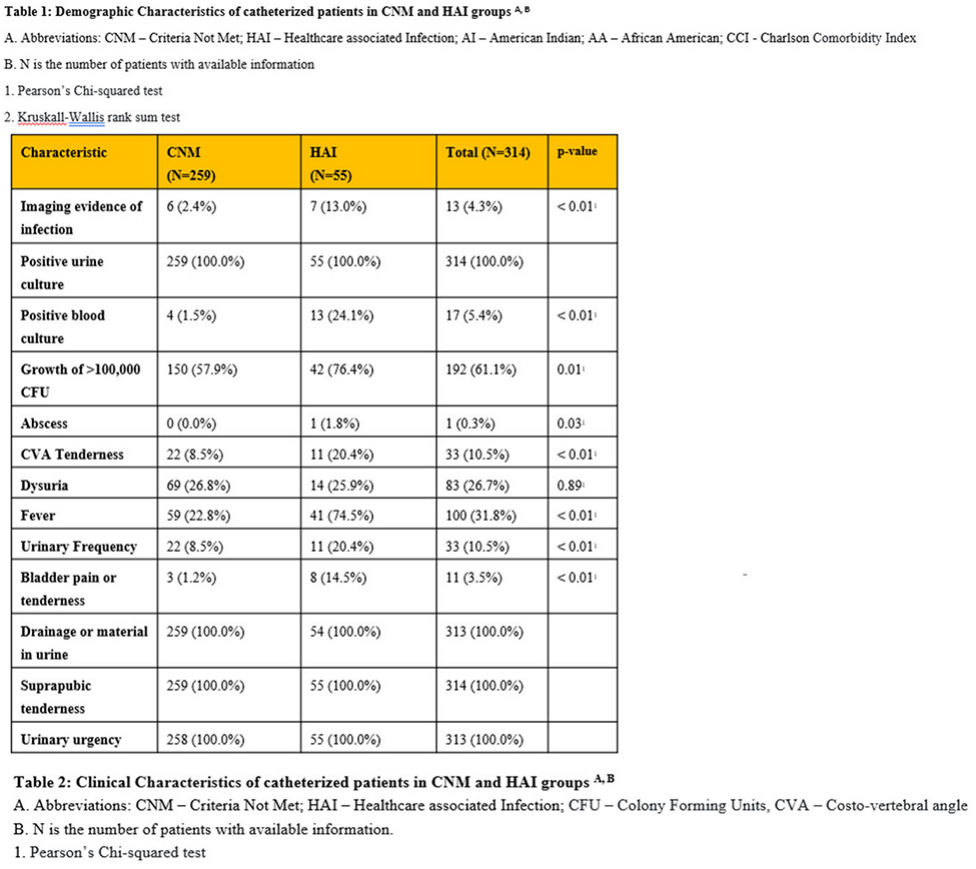

**Methods:**

We conducted a retrospective review of newly catheterized patients at a tertiary hospital over 12-months period. Demographic, clinical, and diagnostic data were collected. Based on the National Health Safety Network (NHSN) criteria, patients were divided into two groups: health care associated infection (HAI) and criteria not met (CNM). Outcome measures included secondary bloodstream infections, hospital re-admission, death, and microbiological relapse at 30 days of hospital discharge. These outcomes were compared between groups.

**Figure:**
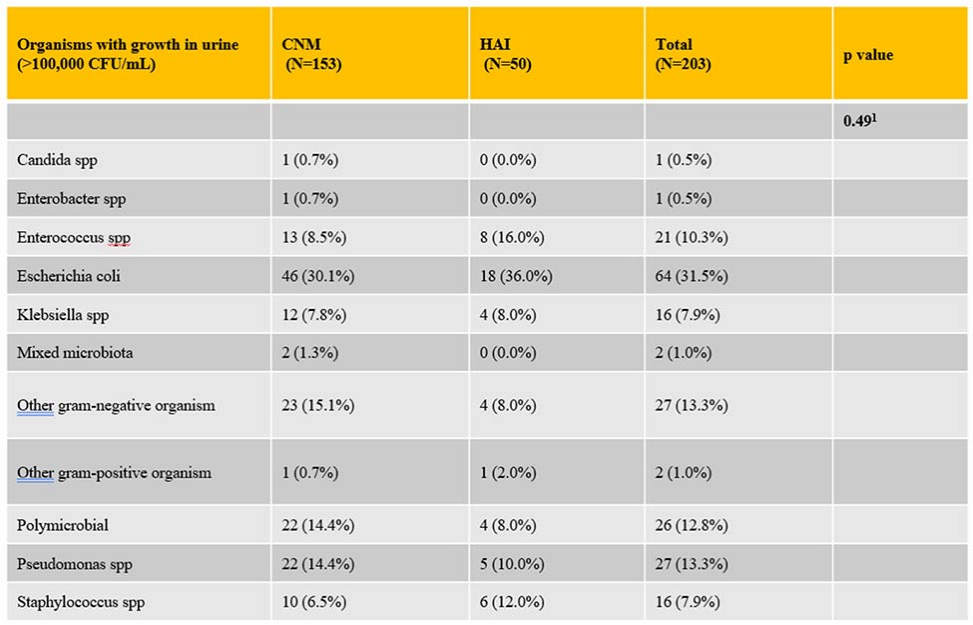

**Figure:**
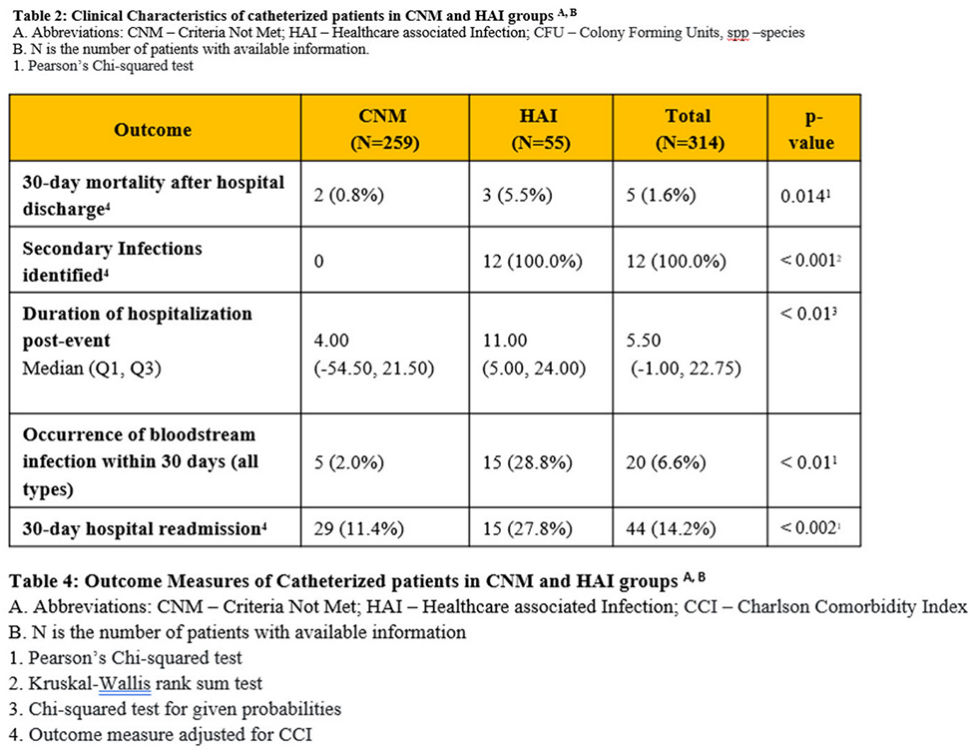

**Results:**

A total of 314 patients were identified, of whom 55 (17.5%) met the criteria for HAI. Demographic, clinical and microbiological data are summarized in Tables 1-3. In comparison to the CNM group, the HAI group showed higher rates of fever, urinary frequency, bladder pain or tenderness, costovertebral angle tenderness, positive blood cultures, growth of urinary microorganisms >100,000 colony forming units (CFU), and imaging evidence of infection (p< 0.01). Blood stream infections were observed in 20 (6.6%) patients, with 12 of these in the HAI group, and were attributed to a urinary source in 7 cases. Composite outcomes were affected by fever (p< 0.01), imaging evidence of infection (p< 0.01) and presence of renal abscess, whereas the age of catheter, dysuria, bladder tenderness, urinary frequency, urinary CFU threshold didn't achieve statistical significance. After adjusting for the Charlson Comorbidity Index, the HAI group showed higher rates of re-occurrence of blood stream infections, re-admission and mortality within 30 days (Table 4).

**Conclusion:**

Fever, imaging evidence of infection, and renal abscess were significant predictors of adverse composite outcomes, whereas catheter age, dysuria, bladder tenderness, urinary frequency, and urinary CFU threshold were not. Refinement of the NHSN surveillance definition of CAUTI may enhance targeted resource allocation for prevention efforts.

**Disclosures:**

All Authors: No reported disclosures

